# Editorial: Exosomal biomarkers: Roles in diagnostics and therapeutics

**DOI:** 10.3389/fmolb.2022.1127540

**Published:** 2023-01-11

**Authors:** Dwijendra K. Gupta

**Affiliations:** Department of Biochemistry, Allahabad University, Allahabad, India

**Keywords:** exosomes, biomarker, therapeutics, methods, tools

Exosomes have gained recognition due to their pivotal role in both intercellular and intracellular communication and in transport of diverse key biomacromolecules. The fact that exosomes appear in almost all biological fluids, including plasma, bronchial fluid, cerebrospinal fluid (CSF), and breast milk (to name a few), has attracted the attention of biologists working in various areas of science.

For this Research Topic, 12 submissions were received, of which eight articles were accepted by the Frontiers editorial team. These consisted of three original research articles, four reviews, and one brief research report. The accepted submissions represent a significant contribution to the advancement of exosome and exosome-based diagnostics, non-invasive biopsy, and therapeutic approaches. The published articles are of high quality and add new information to this fast-growing area of research, adopting a futuristic approach toward non-invasive diagnosis and novel therapeutic strategies.

In search of surface or surface-associated proteins on extracellular vesicles (EVs) that can serve as diagnostic biomarkers, the research group of Moller Jorgensen (Clegg et al.) developed a simple and elegant multi-parametric microarray-based procedure, termed EV Array, that enables the measurement of multiple EV marker proteins in a single analysis. This method uses extremely small amounts of body fluids, directly obviating the need for time-consuming and tedious purification protocols. The authors report that their method is being used in the hospitals of Aalborg University for several clinical diagnostic and prognostic studies involving diseases such as lung cancers, multiple sclerosis, and atrial fibrillation. Certainly, investigators will find the EV Array procedure helpful in assessing the expression, if any, of EV markers in clinical situations and in making quick diagnoses.

A research group led by Ye and Liu selected a unique Research Topic within the area of EV research, presenting an interesting review of the roles of macrophage-derived extracellular vesicles (EVs) in multisystem diseases, with particular focus on cancers, cardiovascular diseases, inflammatory diseases, and osteo-related pathologies. Another interesting component of this review relates to the bioengineering of macrophage EVs and their usefulness as biomarkers in disease diagnosis and treatment.

Another group, that of Gulati et al., reviews published research suggesting theranostic applications for exosomes in malignancies and their potential to help in the assessment of the clinical prognosis of aggressive cancers. The authors also review the role of exosomes in various signaling mechanisms, including in angiogenesis and tumor metastasis, immuno-editing, and drug resistance.

In yet another article, Zhang et al. review the latest advancements in the use of EVs as prognostic and diagnostic biomarkers and as therapeutic agents, and discuss specific diagnostic or therapeutic applications of EVs as novel drug delivery vehicles, as well as their applications in treatment of cardiovascular diseases. The authors also discuss current perspectives, challenges, and potentialapplications in terms of the clinical and industrial transformation of EVs.

An interesting submission is also presented on the role of exosomes in COVID-19. In the context of the emergence in late 2019 of the new SARS-CoV-2 virus, which affected lives worldwide, including those of pregnant women, Y Singh’s group (Cao et al.) investigated the available range of approaches in relation to prevention, diagnostics, and therapeutics. Specifically, they investigated how COVID-19 affects the production of exosomes in pregnant women who have recovered from this pandemic disease and how these exosomes regulate the adaptive immune response. In their study, the authors report observing several exosomal markers (including CD9, CD31, CD40, CD45, CD41b, CD42a, CD62P, CD69, CD81, CD105, and HLA-DRDPDQ) to be significantly less abundant in the plasma of COVID-19-recovered pregnant women compared with a control group, thus concluding that COVID-19-recovered pregnant women possess less immunogenic protection.

Feng et al. studied a complex multifactorial disease model, namely intervertebral disc (IVD) degeneration, with ill-defined pathogenesis. They investigated whether exosomes derived from degenerative nucleus pulposus cells (NPCs) promote apoptosis of cartilage endplate (CEP) cells and aggravate IVD degeneration, there being few existing studies on the information interaction between NPCs and CEP cells. Their study demonstrates that degenerated NPC-exosome could indeed induce apoptosis of CEP cells, inhibit ECM synthesis, and promote ECM degradation. In addition, their findings indicate that degenerated NPC-exosome aggravates IVD degeneration.

In their excellent review, Zeng et al. discuss the advantages of and the challenges associated with the applications of extracellular vesicles (EVs), which are now known to be involved in a variety of pathophysiological processes in many diseases. They also discuss potential resolutions for the shortcomings of the relevant isolation and purification methods, storage conditions, and pharmacokinetics and biodistribution patterns during drug delivery, which will certainly facilitate the clinical application of EVs. However, due to size differences and a lack of specific markers, both EVs and exosomal preparations are heterogeneous, with questionable purity and origin. The position of the International Society for Extracellular Vesicles, based in Gothenburg (Switzerland), is that *EVs* is a preferable term to *exosomes* and that the former should be adopted, although these terms are used interchangeably by several groups.

Finally, a group led by Hong Wu et al. reports on their research, in the form of a bioinformatics-based study, on the diagnostic application of exosomes in hepatocellular carcinoma (HCC), which is globally considered to be among the fifty most common cancers, with a high mortality rate, representing the second most common cause of cancer-related deaths. The researchers accessed exosome data from the exoRBase database and a free online database to estimate potential binding miRNA of mRNA, lncRNA, and circRNA, and to identify useful exosome biomarkers for HCC therapy. Their transcriptome study using Cytoscape revealed 159 mRNAs, 60 lncRNAs, and 13 circRNAs to be differentially expressed; additionally, March8, SH3PXD2A, has-circ-0014088, hsa-miR-186-5p, and hsa-miR-613 were identified as hub biomarkers. Furthermore, their KEGG pathway analysis demonstrated the differentially expressed proteins to be principally involved in the MAPK signaling network, central carbon metabolism in cancer, the glucagon signaling pathway, glutamatergic synapse, and spliceosome. Finally, an evaluation of the protein expression of SMARCA5, CDC42, and UBC in normal vs. cancer tissues using the Human Protein Atlas (HPA) online tool showed that the expression of these three genes is significantly upregulated in tumor tissues. This could mimic exosome micro-environmental disorders, providing potential biomarkers for therapeutic applications of exosomes.

